# Musculoskeletal involvement in childhood leukemia: Characteristics and survival outcomes

**DOI:** 10.1186/s12969-022-00692-9

**Published:** 2022-05-02

**Authors:** Sirinthip Kittivisuit, Pornpun Sripornsawan, Natsaruth Songthawee, Shevachut Chavananon, Edward B. McNeil, Thirachit Chotsampancharoen

**Affiliations:** 1 Department of Pediatrics, Faculty of Medicine, Prince of Songkla University, Hat Yai, Thailand; 2 Epidemiology Unit, Faculty of Medicine, Prince of Songkla University, Hat Yai, Thailand

**Keywords:** Acute leukemia, Musculoskeletal involvement, Survival outcome

## Abstract

**Background:**

Childhood leukemia with musculoskeletal (MSK) involvement mimics various conditions, which consequently leads to diagnostic delays. The clinical implication of MSK involvement in this disease on survival outcomes is inconclusive. This study aimed to compare characteristics and survival outcomes between MSK and non-MSK involvement in childhood leukemia.

**Methods:**

The medical records of children newly diagnosed with acute leukemia of an age under 15 years were retrospectively reviewed. Two-to-one nearest-neighbor propensity score-matching was performed to obtain matched groups with and without MSK involvement. The Kaplan–Meier method and log-rank test were then used to assess the effect of MSK involvement on survival outcomes.

**Results:**

Of 1042 childhood leukemia cases, 81 (7.8%) children had MSK involvement at initial presentation. MSK involvement was more likely in children with acute lymphoblastic leukemia than acute myeloid leukemia (*p* < 0.05). Hematologic abnormalities were less frequent in the MSK involvement group (*p* < 0.05). The absence of peripheral blast cells was significantly higher in the MSK involvement group (17.3% vs 9.6%, *p* = 0.04). Normal complete blood counts with absence of peripheral blast cells were found 2.5% of the children with MSK involvement. By propensity score-matching for comparable risk groups of children with and without MSK involvement, the 5-year overall survival was not significantly different (48.2% vs 57.4%, respectively, *p* = 0.22), nor was event-free survival (43.3% vs 51.8%, respectively, *p* = 0.31).

**Conclusion:**

Childhood leukemia with MSK involvement had the characteristics of minimal or absent hematologic abnormalities and peripheral blast counts.

## Background

Acute leukemia is the most common malignancy of childhood, accounting for one-third of childhood malignancies, with age-standardized incidence rates of 36.1–46.4 per million person-years [1, 2]. Apart from the characteristic symptoms of fever, pallor, bleeding tendency, hepatomegaly, and splenomegaly, there are a number of unusual clinical manifestations including musculoskeletal (MSK) symptoms [3]. MSK involvement has been reported in childhood leukemia at rates varying from 7.1 to 62.3% [4–12]. In 2016, a systematic review and meta-analysis reported that MSK symptoms were a prominent clinical presenting feature in childhood leukemia, including limb pain (43%), bone pain (26%), joint pain (15%), and limping (11%) [3]. Various special characteristics of childhood leukemia with MSK involvement have been reported in the literature. For example, in some studies, MSK involvement was found to be more frequent in acute lymphoblastic leukemia (ALL), especially B-cell ALL, than acute myeloid leukemia (AML) [13–15]. This subgroup with MSK involvement also had lower rates of hematologic abnormalities, as well as peripheral blast counts at initial presentation [12–16]. Therefore, childhood leukemia with MSK involvement can mimic rheumatic or orthopedic conditions and lead to delayed diagnosis of leukemia [14, 16, 17]. To date there is no consensus on the prognostic significance of MSK involvement in childhood leukemia. In consideration of the limited data and the uncertain prognosis of childhood leukemia with MSK involvement, this study aimed to identify the clinical and laboratory characteristics at initial presentation of childhood leukemia with MSK involvement, and to compare survival outcomes between children with and without MSK involvement.

## Methods

We retrospectively reviewed the medical records of 1221 children aged < 15 years diagnosed with acute leukemia between January 1978 and December 2019, and treated at the Oncology Clinic, Department of Pediatrics, Faculty of Medicine, Prince of Songkla University, which is the major tertiary-referral center in southern Thailand. The information recorded for each child included demographic characteristics, clinical manifestations at diagnosis, and initial laboratory investigations such as complete blood count (CBC), blood chemistry, subtype of acute leukemia (ALL or AML) and year of diagnosis. Diagnosis of AML or ALL and subtype was made according to the French-American-British (FAB) classification by morphological examination of bone marrow staining in all patients. Cytochemical stains included periodic acid-Schiff, peroxidase and α-naphthyl acetate esterase. After the year 2000, immunophenotyping of cell surface markers was additionally used to differentiate subtypes (i.e., AML from ALL, and T-cell ALL from B-cell ALL). The National Cancer Institute (NCI) risk classification was used to separate high and standard risk groups based on age and white blood cell (WBC) count at diagnosis. We used the standard NCI risk classifications, in which patients aged 1 to 10 years with WBC values less than 50 × 10^9^/L were defined as low risk, while the rest were defined as high risk [18]. Prior to 2006, our treatment chemotherapy protocols were based on the modified ALL treatment protocols of the Children’s Cancer Study Group [19–21] and the AML treatment protocols of the Berlin-Frankfurt-Munster group [22–24]. In 2006, the Thai Pediatric Oncology Group developed the national standardized protocols for treatment of childhood leukemia in Thailand [25]. After that our treatment protocols were based on the national protocols according to the subtypes of leukemia and risk stratification of patients at presentation.

The MSK symptoms at initial presentation of each child were reviewed. MSK involvement was defined as bone and/or joint pain (either arthralgia or arthritis). Arthralgia was defined as pain localized in one more joints and arthritis was defined as joint pain with signs of joint inflammation (joint swelling, effusion, redness, increased heat, or limited range of motion). The pattern of joint involvement was defined as monoarticular (one joint), oligoarticular (2–4 joints), and polyarticular (5 or more joints). Initial bone and joint radiographs of all children who had MSK involvement were reviewed by a radiologist for the characteristics of radiographic bone changes in leukemia including metaphyseal radiolucent bands, osteolytic lesions, osteosclerosis, and periosteal reaction [26, 27].

### Statistical analysis

Descriptive statistics are presented using mean and standard deviation or median and interquartile range (IQR) for continuous variables as appropriate, and frequency with percentage for categorical variables. Demographic and clinical characteristics were compared using the chi-square or Fisher’s exact test for categorical variables and Student's *t*-test or rank-sum test for continuous variables as appropriate. Two-to-one nearest-neighbor propensity score-matching without replacement was performed to reduce the imbalance in the distribution of confounding variables between the groups with and without MSK involvement using the MatchIt package in R (R Foundation for Statistical Computing, Vienna, Austria). Matching variables included age, sex, initial WBC count, phenotype of leukemia, and period of diagnosis. Survival analyses were performed using the Kaplan–Meier estimator and log-rank test. All statistical tests were two-sided and p-values < 0.05 were considered statistically significant.

## Results

During the 42-year study period, 1221 newly diagnosed children with acute leukemia were identified. We excluded 87 children who had incomplete data and 92 children who refused standard chemotherapy and were treated with alternative traditional medicine alone, leaving a total of 1042 children for analysis. Of these, 81 (7.8%) were reported as having MSK involvement in various bones and/or joints. The median time (IQR) from the beginning of the MSK symptoms to the time of leukemia diagnosis was 30.0 (21.0–90.0) days. The main clinical presentations of the MSK involvement were joint pain in 95.1% (77/81) and bone pain in 33.3% (27/81). Of the 81 children with MSK involvement, 23 (28.4%) had both joint pain (arthritis or arthralgia) and bone pain. Among the 77 children who had joint pain, 66 (85.7%) had arthritis, while 11 (14.3%) had arthralgia without signs of joint inflammation. The most common pattern of joint pain was polyarticular (46.8%), followed by oligoarticular (29.9%) and monoarticular (23.4%) patterns. The most common sites of joint pain were the large joints of the extremities, notably ankles (58.4%), knees (57.1%), and elbows (46.8%). Among 78 of 81 children with MSK involvement who had radiologic work ups of the site of their MSK involvement at initial presentation, 64 (82.1%) had the characteristic radiographic bone changes of leukemia confirmed by a radiologist. None of the 81 children with MSK involvement were primarily presented to a pediatric rheumatologist and none exhibited persisting symptoms after the induction phase of chemotherapy.

Of the 81 children with MSK involvement, 29 (35.8%) were initially misdiagnosed with other diseases before the diagnosis of acute leukemia. Among the 29 children who were initially misdiagnosed, 17 (58.6%) were diagnosed with juvenile idiopathic arthritis (JIA). Of the 17 children who were initially misdiagnosed with JIA, 14 (82.3%) presented with polyarthritis. Six children were initially diagnosed with systemic JIA. Two children had no other symptoms and hematologic abnormalities besides MSK symptoms. All 17 children received treatment with non-steroidal anti-inflammatory drugs but none of them received corticosteroids or other immunosuppressive drugs before the diagnosis of leukemia. The median time from the beginning of the MSK symptoms to the time of leukemia diagnosis in the 29 children who were initially misdiagnosed was longer than the 52 who were not; however, the difference was not statistically significant (60 days vs 30 days, *p* = 0.07).

The demographic and hematologic characteristics of the 81 children with MSK involvement and the 961 children without MSK involvement at initial presentation are presented in Table 1. The median age at onset among the children with MSK involvement was slightly higher than in those without MSK involvement. The subtype of acute leukemia was significantly different between the children with and without MSK involvement. More children with MSK involvement were diagnosed with ALL (88.9%) than AML (11.1%). More children with MSK involvement were classified as B-cell ALL (69.4%) than T-cell ALL (4.2%). Children with MSK involvement had fever significantly more frequently than those without. Skin bleeding and splenomegaly were significantly less frequent in the group without MSK involvement and hepatomegaly and lymphadenopathy were slightly less frequent in those with MSK involvement, but the differences were not statistically significant.

Regarding the laboratory findings at initial presentation, children with MSK involvement had significantly higher hemoglobin (Hb) and platelet counts, and lower WBC and peripheral blast counts (*p* < 0.05). The absence of peripheral blast cells was significantly higher in the MSK involvement group than in those without MSK involvement (17.3% vs 9.6%, *p* = 0.04). Among 81 children with MSK involvement, 2.5% (2/81) presented with a normal CBC (Hb ≥ 10 g/dL, WBCs 5.0–15.0 × 10^9^/L, platelet count ≥ 100 × 10^9^/L and absence of peripheral blasts). The median lactate dehydrogenase (LDH) level was significantly lower in the MSK involvement group (*p* < 0.05). Other laboratory parameters including serum calcium, phosphate, uric acid, and alkaline phosphatase were not significantly different between the children with and without MSK involvement.

The median survival time for the entire 1042 study patients was 3.23 years and the 10-year overall survival rate was 38.8%. There was no difference in the overall survival (OS) and event-free survival (EFS) rates between the 81 children with MSK involvement and the 961 children without MSK involvement (*p* = 0.22 and 0.17, respectively). Among the 81 children with MSK involvement, the OS and EFS were not significantly different between the 29 children who were initially misdiagnosed with other diseases and the 52 who were not (*p* = 0.40 and 0.47, respectively).

**Fig. 1:**
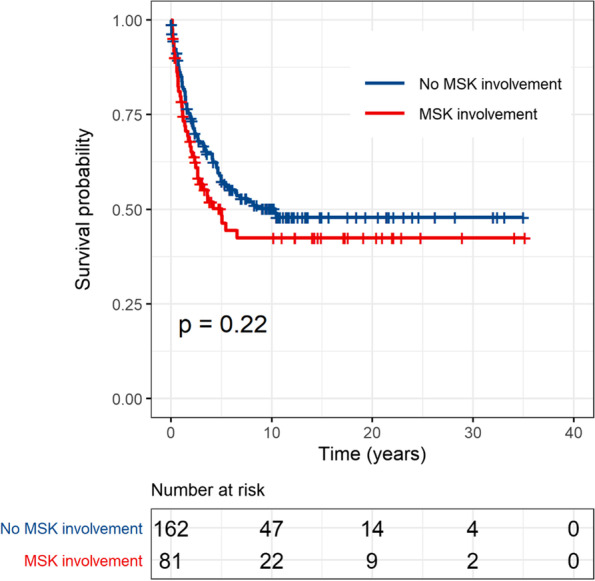
Comparison of overall survival by musculoskeletal involvement among the 243 propensity-score matched study patients

**Fig. 2:**
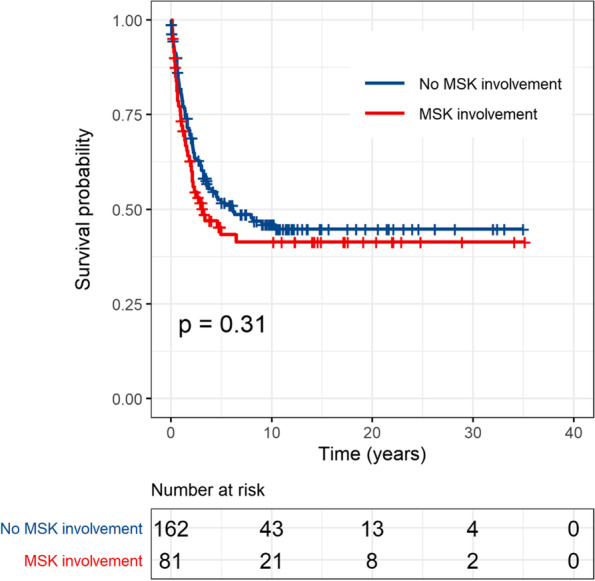
Comparison of event free survival by musculoskeletal involvement among the 243 propensity-score matched patients

Comparisons of the distribution of matching factors and other covariates between groups of children with MSK and without MSK involvement after propensity-matching process are shown in Table 2. There were no differences in the risk variables between the 2 groups. The Kaplan–Meier curves showing the comparisons of survival outcome between the 81 children with MSK involvement and the 162 matched children without MSK involvement are presented in Fig. 1 (overall survival, OS) and Fig. 2 (event-free survival, EFS). The OS and EFS rates were lower for children with MSK involvement compared with the propensity score-matched children without MSK involvement, but the differences were not statistically significant (*p* = 0.22 and 0.31, respectively). The 5-year OS rate for the 81 children with MSK involvement was 48.2% compared with 57.4% for the 162 matched children without MSK involvement. The 5-year EFS rate for the 81 children with MSK involvement was 43.3% compared with 51.8% for the 162 matched children without MSK involvement.

## Discussion

Our long-term study covered a 42-year period and included a total of 1042 childhood leukemia patients. Of these, the incidence of MSK involvement at initial presentation was 7.8%, which was within the ranges previously reported from 7.1 to 62.3% in childhood leukemia [4–12]. These quite disparate ranges may have resulted from the variety of definitions used in previous studies, as some included only cases with bone and/or joint pain while others also included cases with subjective complaints such as non-specific limb pain, refusal to walk, or abnormal gait. Among the studies which included only cases with bone and/or joint pain, Kai et al. found that 18.7% of 225 childhood leukemia patients had symptoms referable to bones and/or joints [4]. Similarly, Biswas et al. reported that 27.3% of their 75 childhood leukemia patients had bone and/or joint pain [9]. Our study reported a lower incidence of MSK involvement in childhood leukemia than these previous studies reporting only cases with bone and/or joint pain. The explanation may be differences in the definitions, as apart from bone and joint complaints, most of the cases in our study had radiologic evidence of bone and joint involvement. In comparison, the studies which included subjective MSK complaints tended to report higher incidences of MSK involvement. Riccio et al. [11] retrospectively reviewed 328 childhood leukemia cases and reported that 22.3% of their cases had MSK symptoms while Robazzi et al. reported that 54.7% of 406 childhood leukemia patients had osteoarticular manifestations [7].

MSK involvement has been reported more commonly in ALL than AML patients, especially in B-cell ALL [4, 7, 13–15]. Similarly, the majority of children who had MSK involvement in our study were in the ALL subgroup. When focusing on a subgroup of childhood ALL, previous studies reported the incidence of MSK involvement ranged from 8.4 to 47.4% [13–17, 28, 29]. Our study found that the incidence of MSK involvement in childhood ALL was 9.9% (72/726), which was within the lower range of previous studies. There are only a limited number of studies investigating MSK involvement in childhood AML. Kai et al. reported that 10.5% of 57 childhood AML cases had MSK involvement [4]. Robazzi et al. investigated 93 childhood AML cases and found arthritis in 9.7% and arthralgia in 5.4% [7]. Our study, which included a total of 316 children diagnosed with AML, found that the incidence of MSK involvement in this subgroup was 2.8%.

Our study found that joint pain was the most common MSK involvement, and the large joints of the extremities, especially the ankles and knees, were the most commonly involved sites. This clinical characteristic of MSK involvement were similarly reported in previous studies [7, 11, 14, 29–31]. The polyarticular joint pain pattern was the most common presentation in our study, which was similar to a study by Spilberg and Meyer [30]. However, most previous studies found that the oligoarticular pattern was the most common presentation [10, 12, 14, 29].

Laboratory characteristics of slight hematologic abnormalities at initial presentation of higher Hb level and lower WBC and higher platelet counts, have been reported in childhood leukemia with MSK involvement compared with patients without MSK involvement [12–16]. Similarly, we found that hematologic abnormalities were significantly less frequent in the group with MSK involvement. Few or even the absence of peripheral blast counts has also been reported as a distinctive finding in childhood leukemia with MSK involvement in previous studies [12, 13, 16]. In the same way, our study found that the absence of peripheral blast cells was significantly higher in the group with MSK involvement. In our study, 2.5% of 81 childhood leukemia with MSK involvement had normal CBCs and absence of peripheral blasts. Similarly, Jonsson et al. found that 3.9% of 52 childhood ALL cases with MSK symptoms presented with normal CBCs and absence of peripheral blasts [16]. Barbosa et al. [5] found that 4.9% of 61 childhood leukemia cases presented with normal CBC while Ma et al. reported that 18% of 225 childhood leukemia cases in their study had minimal anemia and neutropenia with normal platelet counts and absence of peripheral blasts [28].

The prognosis of survival outcomes of MSK involvement in childhood leukemia is controversial. Some studies have reported that children with MSK involvement had better survival outcomes than those without MSK involvement [14, 32–34], while many found no difference in survival outcomes [8, 13, 15, 17, 29, 35, 36] and one study reported a poor prognosis in patients with severe bone involvement [37]. Our study, using a propensity score-matching method for balancing risks between the study groups, found that the presence of MSK involvement did not have a significant association with prognosis in childhood leukemia.

Our long-term study included a large number of childhood leukemia patients. The majority with MSK involvement in our study had their disease confirmed by radiographic bone changes. Our study also had some limitations. First, there is potential bias inherent in any long-term, retrospective study, and although the chart records of all childhood leukemia patients in our center included data of the bone and joint involvement at the initial presentation, subjective complaints such as limb pain, back pain, limp or night pain might have been underreported. Second, MSK symptoms which could have developed later during the disease course were not included in our study. Third, the validity of the study is also limited by factors such as the period of diagnosis where there may have been differences in classification of ALL (from FAB to T-cell or B-cell ALL), the method of diagnosis, which additionally used immunophenotyping of cell markers, and treatment protocols. Fourth, the lack of molecular studies limited the assessment of these variables in our study. Fifth, several related variables, including the initiation of treatment, were not evaluated in our study. Thailand has a limited number of pediatric rheumatologists. In the past, there was no pediatric rheumatologist service in our center. Therefore, none of the children in our study had been evaluated by a pediatric rheumatologist.

In conclusion, childhood leukemia with MSK involvement had the notable characteristics of minimal or absent hematologic abnormalities and peripheral blast counts. The survival outcomes were not different between those with and without MSK involvement. As the clinical and laboratory signs of leukemia can be obscured at initial presentation, clinicians should be aware of the possibility of leukemia in children who present with MSK complaints.

**Table 1:** Comparison of demographic and clinical characteristics between childhood leukemia patients with and without musculoskeletal involvement

**Factor **	**Total** **(*N*=1042)**	**No MSK involvement** **(*N*=961)**	**MSK involvement** **(*N*=81)**	**P value**
Median age, years (IQR)	4.9 (2.9-9.0)	4.9 (2.8-9.0)	5.9 (3.5-9.3)	0.052
Sex				0.787
Male	613 (58.8)	567 (59.0)	46 (56.8)	
Female	429 (41.2)	394 (41.0)	35 (43.2)	
Year of diagnosis				0.036
1979-1999	378 (36.3)	354 (36.8)	24 (29.6)	
2000-2009	413 (39.6)	385 (40.1)	28 (34.6)	
2010-2019	251 (24.1)	222 (23.1)	29 (35.8)	
Morphology				<0.001
ALL	726 (69.7)	654 (68.1)	72 (88.9)	
AML	316 (30.3)	307 (31.9)	9 (11.1)	
ALL subtype				0.028
B-cell	401 (55.2)	351 (53.7)	50 (69.4)	
T-cell	71 (9.8)	68 (10.4)	3 (4.2)	
FAB classification	254 (35.0)	235 (35.9)	19 (26.4)	
Fever				0.005
Yes	770 (73.9)	699 (72.7)	71 (87.7)	
No	272 (26.1)	262 (27.3)	10 (12.3)	
Skin bleeding				<0.001
Yes	444 (42.6)	425 (44.2)	19 (23.5)	
No	598 (57.4)	536 (55.8)	62 (76.5)	
Hepatomegaly				0.149
Yes	909 (87.2)	843 (87.7)	66 (81.5)	
No	133 (12.8)	118 (12.3)	15 (18.5)	
Splenomegaly				0.043
Yes	643 (61.7)	602 (62.6)	41 (50.6)	
No	399 (38.3)	359 (37.4)	40 (49.4)	
Lymphadenopathy				0.359
Yes	808 (77.5)	749 (77.9)	59 (72.8)	
No	234 (22.5)	212 (22.1)	22 (27.2)	
NCI risk group*				0.017
Standard	595 (57.4)	538 (56.0)	57 (70.4)	
High	447 (42.9)	423 (44.0)	24 (29.6)	
Hb, g/dL	7.0 (5.4-8.8)	7.0 (5.3-8.7)	8.0 (6.5-9.2)	0.001
WBC, ×10^9^/L	17.1 (5.9-62.4)	18.1 (6.1-69.2)	9.0 (5.3-22.7)	<0.001
WBC, ×10^9^/L				0.002
<20	557 (53.5)	500 (52)	57 (70.4)	
≥20	485 (46.5)	461 (48)	24 (29.6)	
Platelets, ×10^9^/L	38.0 (18.0-80.0)	36.0 (17.0-73.0)	78.0 (43.0-168.0)	<0.001
Platelets, ×10^9^/L				<0.001
<100	853 (81.9)	804 (83.7)	49 (60.5)	
≥100	189 (18.1)	157 (16.3)	32 (39.5)	
Blasts in peripheral blood, %	50.0 (13.0-83.0)	52.0 (14.8-85.0)	26.0 (5.0-64.0)	<0.001
Blasts in peripheral blood				0.044
Absence	106 (10.2)	92 (9.6)	14 (17.3)	
Presence	935 (89.8)	868 (90.4)	67 (82.7)	
Calcium, mg%	9.4 (8.9-9.8)	9.4 (8.9-9.8)	9.5 (9.0-10.0)	0.104
Phosphate, mg%	4.7 (3.9-5.4)	4.7 (3.9-5.4)	4.7 (4.2-5.3)	0.996
Uric acid, mg%	5.2 (3.9-6.8)	5.2 (4.0-6.9)	5.0 (3.7-6.3)	0.168
ALP, U/L	141.0 (105.0-193.0)	141.0 (106.0-193.0)	135.0(99.8-190.8)	0.725
LDH, U/L	1047.0(628.0-2171.5)	1065.0(652.0-2207.5)	831.0(532.8-1465.2)	0.013
LDH, U/L				0.008
<500	116 (12.9)	99 (12)	17 (23.6)	
≥500	782 (87.1)	727 (88)	55 (76.4)	

Values are expressed as n (%), or median (IQR). ALP; alkaline phosphatase; ALL, acute leukemia; AML; acute myeloid leukemia, FAB, French-American-British classification system; Hb, hemoglobin; LDH; lactate dehydrogenase, MSK, musculoskeletal; NCI, National Cancer Institute; WBC, white blood cell

*The standard NCI risk factors are age 1-10 years with WBC <50×10^9^/L, with the remaining children classified as high NCI risk

**Table 2:** Comparison of characteristics between patients with musculoskeletal involvement and propensity-score matched patients without musculoskeletal involvement

**Factor **	**Total** **(*N*=243)**	**No MSK involvement** **(*N*=162)**	**MSK involvement** **(*N*=81)**	**P value**
Age, years				1
0-10	187 (77)	125 (77.2)	62 (76.5)	
>10	56 (23)	37 (22.8)	19 (23.5)	
Sex				0.891
Male	135 (55.6)	89 (54.9)	46 (56.8)	
Female	108 (44.4)	73 (45.1)	35 (43.2)	
Year of diagnosis				0.981
1979-1999	74 (30.5)	50 (30.9)	24 (29.6)	
2000-2009	83 (34.2)	55 (34)	28 (34.6)	
2010-2019	86 (35.4)	57 (35.2)	29 (35.8)	
Morphology				1
B-cell ALL	150 (61.7)	100 (61.7)	50 (61.7)	
T-cell ALL	9 (3.7)	6 (3.7)	3 (3.7)	
FAB classification	57 (23.5)	38 (23.5)	19 (23.5)	
AML	27 (11.1)	18 (11.1)	9 (11.1)	
WBC, ×10^9^/L				1
<50	27 (11.1)	18 (11.1)	9 (11.1)	
≥50	216 (88.9)	144 (88.9)	72 (88.9)	

Values are expressed as n (%). ALL, acute leukemia; AML; acute myeloid leukemia, FAB, French-American-British classification system; MSK, musculoskeletal; WBC, white blood cell

## Data Availability

The data that support the findings of this study are available on request from the corresponding author. The data are not publicly available due to privacy and ethical restrictions.

## Abbreviations

ALL: Acute lymphoblastic leukemia
AML: Acute myeloid leukemia
CBC: Complete blood count
EFS: Event-free survival
FAB: French-American-British
Hb: Hemoglobin
JIA: Juvenile idiopathic arthritis
LDH: Lactate dehydrogenase
MSK: Musculoskeletal
NCI: National Cancer Institute
OS: Overall survival
WBC: White blood cell
