# Publisher Correction: Musculoskeletal involvement in childhood leukemia: Characteristics and survival outcomes

**DOI:** 10.1186/s12969-022-00695-6

**Published:** 2022-05-23

**Authors:** Sirinthip Kittivisuit, Pornpun Sripornsawan, Natsaruth Songthawee, Shevachut Chavananon, Edward B. McNeil, Thirachit Chotsampancharoen

**Affiliations:** 1 Department of Pediatrics, Faculty of Medicine, Prince of Songkla University, Hat Yai, Thailand; 2 Epidemiology Unit, Faculty of Medicine, Prince of Songkla University, Hat Yai, Thailand

**Correction to: Pediatr Rheumatol 20, 34 (2022)**

**https://doi.org/10.1186/s12969-022-00692-9**

The original publication of this article [1] contained reference to Figs. 1 and 2, but these figures were not included in the article due to an error in the publication process.

**Fig. 1:**
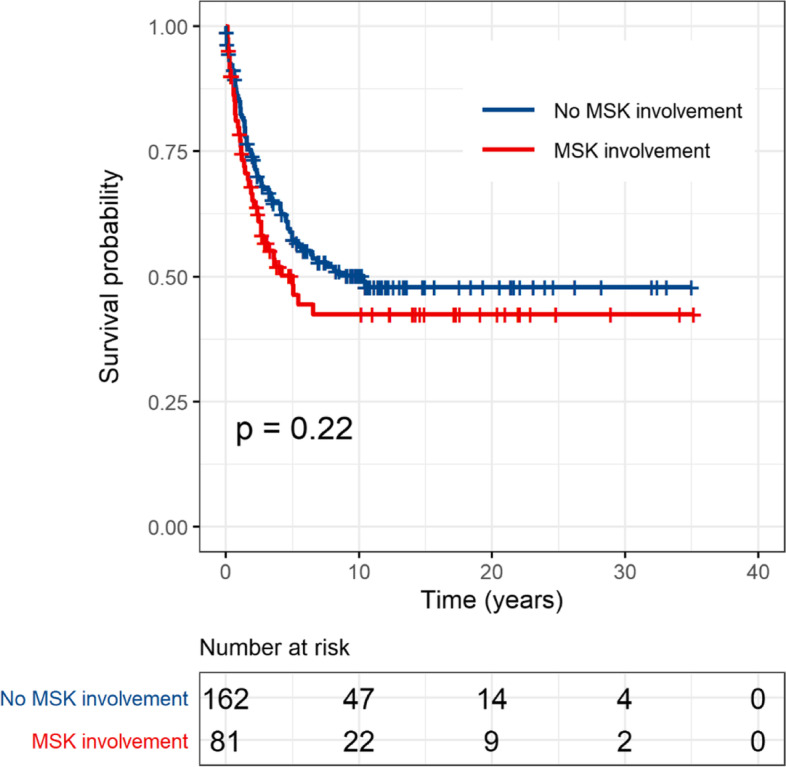
Comparison of overall survival by musculoskeletal involvement among the 243 propensity-score matched study patients

**Fig. 2:**
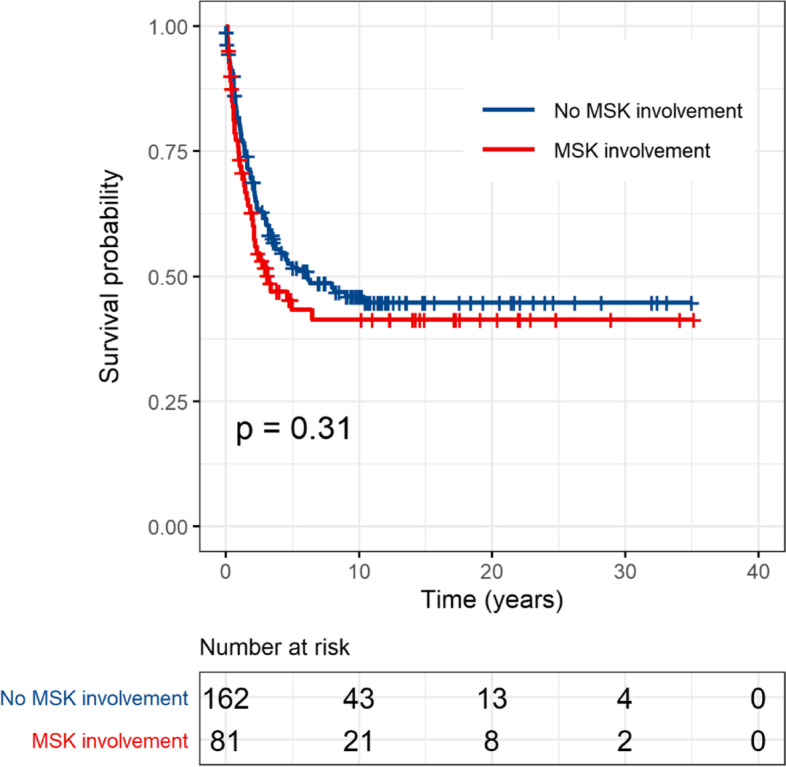
Comparison of event free survival by musculoskeletal involvement among the 243 propensity-score matched patients

The figures are shown in this correction article, the original article has been updated.

The publisher apologizes to the readers & authors for the inconvenience caused.
